# Comparison of Jaw Elevation Device vs. Conventional Airway Assist during Sedation in Chronic Kidney Diseases Undergoing Arteriovenous Fistula Surgery: A Randomized Controlled Trial

**DOI:** 10.3390/jcm10112280

**Published:** 2021-05-24

**Authors:** Sang Hyun Lee, Ji Seon Jeong, Jaeni Jang, Young Hee Shin, Nam-Su Gil, Ji-won Choi, Tae Soo Hahm

**Affiliations:** Samsung Medical Center, Department of Anesthesiology and Pain Medicine, Sungkyunkwan University School of Medicine, Seoul 06351, Korea; shsara2t17.lee@samsung.com (S.H.L.); jiseon78@samsung.com (J.S.J.); jenny.jang@samsung.com (J.J.); yh02.shin@samsung.com (Y.H.S.); ns.gil@samsung.com (N.-S.G.); jiwon0715.choi@samsung.com (J.-w.C.)

**Keywords:** Jaw elevation device, airway, sedation, chronic renal failure, obstructive sleep apnea

## Abstract

Patients with chronic renal failure (CRF) are likely to have obstructive sleep apnea (OSA) underdiagnosed, and maintaining airway patency is important during sedation. This study compared Jaw elevation device (JED) with conventional airway interventions (head lateral rotation, neck extension, oral or nasal airway insertion, and jaw thrust maneuver) during sedation and hypothesized that JED may be effective to open the airway. A total of 73 patients were allocated to a conventional group (*n* = 39) and a JED group (*n* = 34). The number of additional airway interventions was the primary outcome. Percentage of patients with no need of additional interventions and apnea-hypopnea index (AHI) were secondary outcomes. The number of additional interventions was significantly less in the JED group compared to the conventional group (0 (0–0) vs. 1 (0–2); *p* = 0.002). The percentage of patients with no requirement for additional interventions was significantly higher in the JED group compared to the conventional group (76.5% vs. 43.6%; *p* = 0.004). AHI was significantly lower in the JED group compared to the conventional group (4.5 (1.5–11.9) vs. 9.3 (3.8–21.9), *p* = 0.015). In conclusion, JED seems to be effective in opening the airway patency during sedation in CRF patients.

## 1. Introduction

Patients with chronic renal failure (CRF) are known to show a high prevalence of obstructive sleep apnea (OSA), which is often under recognized before surgery [1,2,3,4,5,6,7,8,9,10,11,12]. Arteriovenous fistula (AVF) surgery for these patients can be performed under either general anesthesia (GA) or sedation, with more preference for sedation over GA these days [13]. With a proper use of sedative agents and monitoring, sedation is safe and reliable, but there are concerns about airway management, especially in patients at risk for OSA. Airway neural control and upper airway dilator muscle activity are decreased with sedation [14,15]. Unrecognized OSA can be detrimental to airway patency because it can potentiate snoring and upper airway collapsibility with anesthetic or sedative agents, especially in chronic kidney diseases (CKD) patients because of their increased chemosensitivity [1,16,17,18]. During moderate sedation in CRF patients undergoing AVF surgery, securing the patent airway is of the utmost concern to anesthesiologists, as is at the same time providing adequate analgesia and sedation for the ease of the patient and the surgeon.

Common clinical practice for securing the airway includes head tilt-chin lift, jaw thrust, head lateral rotation and oral airway insertion during sedation [19,20,21,22]. These airway interventions are effective and widely practiced maneuvers to establish the structural patency of the pharyngeal airway, but clinicians often have to be at the head side of the patient with their hands occupied for airway control throughout the procedure. Frequent airway interventions during surgery may impede surgeons’ ease during surgical procedures, and sedation depth may have to be adjusted to near awake condition causing patient discomfort.

Jaw elevation device (JED™; Hypnoz Therapeutic Devices, San Diego, CA, USA) is a FDA (Food and drug administrated) approved, commercially available, externally placed, noninvasive device that assists anesthesiologists to maintain airway patency by lifting the mandible with an artificial jaw thrust arm. This device can be applied to sedated patients in supine position. A recent case report stated that JED is a practical device for reliable ventilation during neuroangiography and thrombectomy under sedation, and it was also effective after intervention for neck immobilization and airway opening [23].

We hypothesized that with an aid of JED, we would reduce the number of additional airway interventions required during sedation in CKD patients compared to the conventional airway intervention maneuvers. Portable polysomnography (PSG) was also performed during sedation to assess for the differences in the degree of apnea-hypopnea index between the JED and conventional airway interventions. The purpose of this study was to determine the usefulness of the JED as an alternative option for maintaining the airway during sedation in CKD patients undergoing AVF surgery.

## 2. Materials and Methods

This randomized controlled study was conducted at the Samsung Medical Center in South Korea between June 2017 and December 2020. All patients provided written informed consent. Adults aged above 20 years diagnosed with CRF, who were scheduled to receive AVF surgery under sedation with propofol and remifentanil between July 2017 and December 2020 were enrolled. Exclusion criteria were allergy to propofol, remifentanil or midazolam; history of central apnea; chronic use of antidepressants, opioids or analgesics; severe respiratory diseases; inability to extend or rotate neck; neurologic dysfunction; and temporomandibular joint dysfunction. Randomization was undertaken with a computer generated randomization table, and patients were allocated to apply the conventional airway interventions or Jaw elevation device (JED™; Hypnoz Therapeutic Devices, San Diego, CA, USA). JED consists of a head support with memory foam and a jaw elevating apparatus at each side with a cushion at the surface touching the skin. All patients were asked to complete a STOP-BANG questionnaire preoperatively, which has been suggested by previous studies as an effective screening tool for sleep apnea [24,25,26,27,28]. STOP-BANG questionnaire involves 8 questions with “yes” or “no” answers (1 point each for “yes”; a sum of “yes” 0–2, low risk; 3–4, intermediate risk; 5–8, high risk of sleep apnea). Questions, which are acronyms for STOP-BANG, are as follows: (1) Snoring while sleeping? (2) Tiredness during daytime? (3) Observed breathing stoppage? (4) Pressure: high blood pressure? (5) BMI > 35 kg/m^2^? (6) Age > 50? (7) Neck circumference > 40 cm? (8) Gender: male?

**Figure 1:**
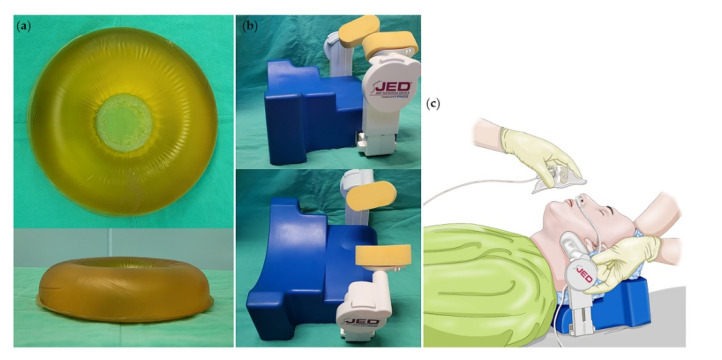
(**a**) Doughnut head gel pad (**b**) Jaw elevation device (JED) (**c**) portable polysomnography nasal cannula applied at the nares and facial mask on top.

Anesthesia monitoring of electrocardiography, pulse oximetry, noninvasive blood pressure, heart rate, respiratory rate and sidestream end tidal carbon dioxide (ETCO_2_) sensor at the nares were recorded at the operating room. Type 3 portable polysomnography (PSG), Embletta MPR^®^ (Embla Systems LLC, Broomfield, CO, USA), was applied at the patient’s nares (nasal prong) before induction, and 6 L/min of oxygen was supplied via facial mask. Facial mask was applied by an elastic bandage (Figure 1). Anesthesia was started and kept constant using the target controlled infusion device (Orchestra^®^; Fresenius Vial, Brezins, France) at effect site concentrations (Ce) of 1.0–3.0 μg/mL for propofol, and 1.0–2.0 ng/mL for remifentanil. Sedation was adjusted to achieve the modified observer’s assessment sedation scale (MOASS) of 2–3. MOASS consists of 0–5; MOASS 0 was no response after painful trapezium squeeze, 1 was response only after painful trapezius squeeze, 2 was response only after mild prodding or shaking, 3 was response only after name is called loudly and/or repeatedly, 4 was lethargic response to name spoken in normal tone, and 5 was response readily to name spoken in normal tone.

At induction, baseline airway patency was confirmed by the attending anesthesiologist. Patients in conventional group were placed in a neutral position with a doughnut gel head pad (height of 4.5 cm, diameter of 20 cm, Figure 1) at the occiput, and patients’ head rotation, neck extension or airway insertion was applied at the discretion of clinicians. In JED group, patients were placed in a neutral position with JED head support (height of 6 cm) at the occiput and jaw elevating apparatus were applied at the temporomandibular joint angle to lift the jaw (Figure 1). After induction, airway modifications were added if the patient showed clinical signs of airway depression or obstruction such as respiratory rate <8/min or apnea detected by ETCO_2_ ≥ 8 s. The types of airway interventions were selected at the clinician’s discretion. Airway intervention types were as follows: (1) head lateral rotation, (2) neck extension, (3) oral airway insertion, (4) nasal airway insertion, (5) jaw thrust maneuver.

At the end of surgery, patients were awakened to the MOASS scale of 4–5 and transferred to the postanaesthesia care unit (PACU). At PACU, patients were questioned about sedation satisfaction using a visual analogue scale (VAS; the number scale from 0 to 10, 0 as no satisfaction and 10 as very much satisfactory) and the presence of pain at the jaw. We also inspected for color change at the jaw.

Rescue interventions for in case of desaturation <93% during sedation was termination of the study and airway access with mask ventilation or supraglottic airway device insertion.

Intraoperative parameters including heart rate, mean blood pressure, respiratory rate, pulse oximeter (SpO_2_), and infused doses of remifenanil and propofol were recorded. PSG related parameters were retrieved from the Embletta MPR device using RemLogic™ (Embla, Thornton, CO, USA) polysomnography software. These parameters included analysis time, apnea-hypopnea index (AHI), total duration and percentage snoring time, and average SpO_2_. In PSG analysis, apnea was defined as breathing cessation lasting for 10 s or more, and hypopnea was defined as a fall of 30% or more of nasal respiratory flow signal. AHI was defined as the number of apneic and hypopneic events per hour of recording time. Snoring was recorded by a microphone assembled in the Embletta MPR device if 3 or more snoring was detected, and snoring periods were merged into one if the interval between them was less than 10 s.

Our primary outcome was the number of additional airway interventions required in each patient during the maintenance of sedation after induction. If more than one type of airway intervention was applied simultaneously on a single occasion, then it was counted as one. Secondary end points were the percentage of patients with no requirement for additional airway interventions, types of airway interventions used, intraoperative sedation parameters, and PSG data.

Sensitivity analysis according to a STOP-BANG score of 0–4 (low to intermediate risk of OSA) vs. 5–8 (high risk of OSA) was performed to compare the number of additional airway interventions and AHI between the groups.

We expected that the number of airway interventions in the JED group would be reduced by at least 50% compared with the conventional group. Sample size was calculated so that at least 34 patients were needed in each group to detect a percentage difference in the number of airway interventions of 50% with a standard deviation of 2 with a power of 80%, α = 0.05. nQuery Advisor 4.0 software was used for the Mann–Whitney U test based on independent two sample t test for sample size calculation. Based on the estimated drop of 20%, recruitment was targeted to be 43 patients in each group. PSG data of a total of 6 patients (3 patients in each group) were missed because of a monitoring error. Therefore PSG data were analyzed excluding these missing data.

Statistical analysis was performed using SPSS statistics 21.0 (IBM, Armonk, NY, USA). Data were presented as mean ± SD or median (interquartile range; IQR) for continuous variables and the number (%) for categorical variables. Continuous parameters were compared with t test or Mann–Whitney U test as appropriate. The normal distribution of the continuous variables was analyzed using the Shapiro–Wilk test. Categorical parameters were analyzed using the chi square test or Fisher’s exact test; *p* <0.05 was considered statistically significant.

## 3. Results

**Figure 2:**
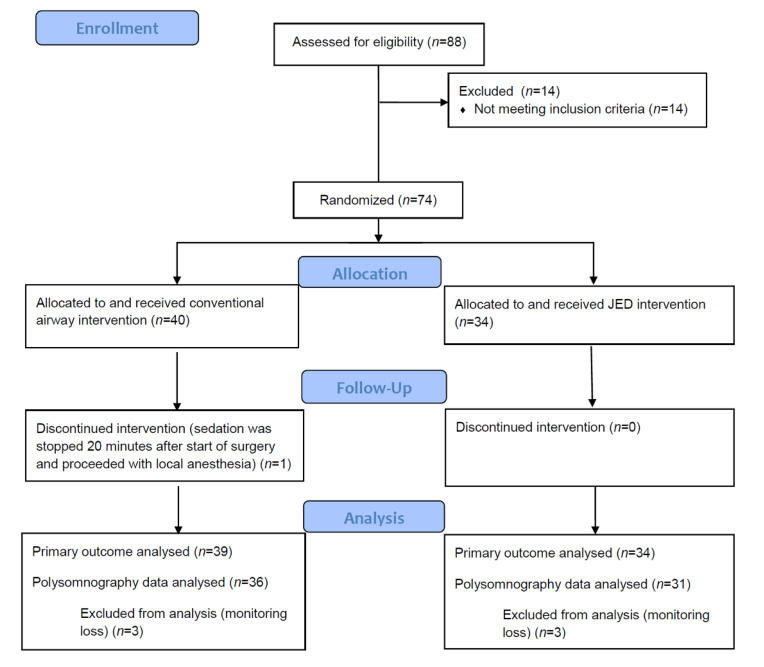
CONSORT flow diagram.

Eighty-eight patients were assessed for eligibility, and 74 patients were finally enrolled. One patient in the conventional group was dropped because sedation was terminated early during surgery and the surgery proceeded with local anesthesia. Thus, 73 patients completed the study for the primary outcome. A CONSORT flow diagram is shown in Figure 2. 

Patient characteristics and preoperative survey data were similar between the groups (Table 1).

Intraoperative sedation data and airway intervention parameters are shown in Table 2 and Table 3. Our primary outcome showed that the number of additional airway interventions was significantly less in the JED group compared to the conventional group (0 (0–0) vs. 1 (0–2); median difference, 1; 95% confidence interval (CI), 0–1; *p* = 0.002). The percentage of patients in each group with no requirement for additional airway interventions was significantly higher in the JED group compared to the conventional group (76.5% vs. 43.6%; difference in proportion, 33%; 95% CI, 10–51%, *p* = 0.004). Among the types of airway interventions, head lateral rotation and neck extension were significantly higher in the conventional group compared to the JED group (*p* < 0.001, *p* = 0.032).

PSG data are shown in Table 4. AHI was significantly lower in the JED group compared to the conventional group (4.5 [1.5–11.9] vs. 9.3 [3.8–21.9]; median difference, 5; 95% CI 1–11; *p* = 0.015). Autonomic arousal index and relative snoring time were statistically not different between the groups. None of the patients complained of jaw pain after surgery, and they were all satisfied (VAS of 10) with anesthesia in both group. None of the patients showed desaturation.

Sensitivity analysis in patients with STOP-BANG of low to intermediate risk (score of 0–4) showed that the number of additional airway manipulation was significantly lower in the JED group compared to the conventional group (0 (0–0) vs. 1.0 (1–2.0); median difference, 1; 95% CI 0–1; *p* = 0.003). AHI showed no statistical difference between the groups (JED group vs. Control group, 2.9 (1.5–8.7) vs. 8.2 (2.8–21.0); median difference, 4; 95% CI, 0–10; *p* = 0.075).

## 4. Discussion

Our results showed that with an application of JED during sedation, the number of additional airway interventions was less frequently required. Indeed, a significantly greater percentage of patients did not require additional airway interventions at all in the JED applied group compared to the conventional airway management applied group. We think it is a clinically useful finding that an externally applied, noninvasive device, JED, is favorable in airway management during sedation. AHI, a highly validated index for assessing OSA [29,30], showed that AHI was significantly lower during sedation in the JED group. Although statistically not significant, total duration and a percentage of snoring time (relative snoring time against analysis duration) were shorter in the JED group. Therefore, JED seems to be effective in opening and maintaining the structural pharyngeal airway during sedation. Association and pathophysiology of developing sleep apnea in CKD patients are still under investigation and are thought to have bidirectional relationship between sleep apnea and CKD [12]. Proposed mechanisms for the role of CKD in developing OSA and central sleep apnea are rostral volume shift, hypervolemia leading to ventilator instability, increased airway collapsibility, and increased chemosensitivity [12].

Interestingly, sensitivity analysis showed that in patients with a STOP-BANG score of low to intermediate risk of OSA (score of 0–4), the JED group showed significantly fewer additional airway interventions compared to the conventional group. The patients in the two groups had shown a similar preoperative STOP-BANG score. JED seems to be effective in fairly low to intermediate risk of OSA patients in securing the airway. But in patients with higher risk of OSA (high STOP-BANG score), JED alone, which mainly elevates the mandible jaw to mechanically open the pharyngeal space, is not as effective as in patients with low to intermediate risk of OSA.

The types of additional airways applied with JED were oral airway insertion or jaw thrust maneuver. In the conventional group, head lateral rotation, neck extension or oral airway insertion were the most frequently used airway interventions. We believe that, along with fewer airway interventions, JED can also free up the hands of the anesthesiologist manually maintaining jaw thrust or head turning and provide comfort to the patients. It could help the surgeons’ procedural convenience and reduce the chance of converting to general anesthesia with airway compromise during a procedure. The benefits of JED may render its use appropriate during procedural sedations in non-operating room anesthesia (NORA), the emergency department (ER) or intensive care unit (ICU).

Portable PSG is a validated examination of recording of nasal airflow via nasal cannula and oxygen saturation via pulse oximetry [29,31]. In previous studies, perioperative portable PSG was used during sedation under spinal anesthesia to detect OSA by the parameter of AHI [30,32]. We also used AHI and snoring time for secondary outcomes to identify the JED effect objectively on airway patency during sedation.

Limitations in our study include the followsing: First, preoperative PSG was not performed, instead we carried out a preoperative sleep apnea (STOP-BANG) questionnaire for all patients, and they were similar between the groups. The primary goal of the study was not to determine absolute sleep apnea status in CRF patients, but to compare the effect of JED during sedation. Second, we were not able to blind the clinicians to group allocation because either use of gel pad or JED was obviously seen. However, additional airway interventions were made by a clinician not involved in the study, and data analysis were also performed by a person not involved in the intervention. Third, sedation depth was monitored by a frontal electroencephalogram, and the BIS value ranges were quite wide. However, all patients were sedated according to the MOAAS at the discretion of clinicians. Fourth, the portable PSG measured the obstructive apnea, so we were not able to differentiate central apnea that may be associated with sedative drugs. During sedation monitoring, patients’ respiratory rate and sidestream ETCO_2_ were used to adjust the sedative agents to prevent severe respiratory depression.

## 5. Conclusions

In conclusion, we were able to use less additional airway interventions by using JED, with lower AHI values during sedation. The externally applied jaw thrust device, JED, seems to be an effective aid to open airway patency compared to conventional airway manipulation during propofol and remifentanil sedation in CRF patients undergoing AVF surgery. With JED, both patient and clinicians benefit clinically with better safety and quality of sedation.

## Figures and Tables

**Table 1:** Patient characteristics and preoperative survey outcomes.

	Conventional Group *n* = 39		JED Group *n* = 34		*p* Value
	Median (IQR)	95% CI	Median (IQR)	95% CI	
Age (year)	64.0 (55.8–71.5)	61.9–69.0	61.5 (54.0–67.0)	55.0–65.0	0.234
Weight (kg)	64.7 (58.5–70.7)	60.5–69.3	66.2 (55.5–71.7)	58.9–69.2	0.867
Height (cm)	165.7 (159.1–168.8)	160.0–168.0	167.5 (160.0–171.0)	164.5–170.0	0.279
Male	25 (64.1%)		23 (67.6%)		0.808
Diabetes Mellitus (%)	22 (56.4%)		17 (51.5%)		0.813
Hypertension (%)	33 (84.6%)		27 (79.4%)		0.760
History of obstructive sleep apnea (%)	4 (10.3%)		6 (17.6%)		0.499
STOP-BANG score ^1^	4.0 (2.5–5.0)	3.0–4.1	3.5 (3.0–5.0)	3.0–4.2	0.860

Numbers are median [IQR, interquartile range] or numbers of patients (percentage) in each group. 95% CI, 95% confidence interval. % percentage of patients in each group. ^1^ Acronym for STOP-BANG: (1) Snoring while sleeping? (2) Tiredness during daytime? (3) Observed breathing stoppage? (4) Pressure: high blood pressure? (5) BMI > 35 kg/m^2^? (6) Age > 50? (7) Neck circumference > 40 cm? (8) Gender: male? Eight questions with “yes” or “no” answers (1 point each for “yes”; a sum of “yes” 0–2, low risk; 3–4, intermediate risk; 5–8, high risk of sleep apnea).

**Table 2:** Intraoperative sedation data.

	Conventional Group *n* = 39		JED Group *n* = 34		*p* Value
	Median (IQR)	95% CI	Median (IQR)	95% CI	
Surgery duration (min)	83.0 (73.8–100.0)	77.9–91.1	88.0 (77.0–97.0)	81.7–92.5	0.756
Intravenous crystalloid fluid (mL)	170.0 (150.0–250.0)	150–250	200.0 (150.0–300.0)	150.0–300.0	0.528
Midazolam (mg)	1.0 (1.0–1.0)	0.0–1.0	1.0 (1.0–1.0)	0.0–1.0	0.507
Hourly infused propofol (mg)	2.49 (2.08–3.12)	2.21–2.82	2.66 (2.02–3.12)	2.14–3.01	0.748
Hourly infused remifentanil (mcg)	1.92 (1.54–2.38)	1.66–2.10	2.07 (1.61–2.54)	1.74–2.31	0.494
Bispectral index (BIS) (*n*)					
Value ranges 60–80	29 (74.4%)		23 (67.6%)		
Value ranges/40–60	9 (23.0%)		11 (32.4%)		
BIS fail	1 (2.6%)		0 (0%)		0.436

Numbers are median [IQR, interquartile range] or numbers of patients (percentage) in each group. 95% CI, 95% confidence interval. *p* value < 0.05 is considered significant.

**Table 3:** Airway interventions during sedation maintenance.

	Conventional Group *n* = 39		JED Group *n* = 34		*p* Value
	Median (IQR)	95% CI	Median (IQR)	95% CI	
Number of additional airway interventions in each patient, *n*	1 (0.0–2.0)	0–1.0	0 (0.0–0.0)	0.0–0.0	0.002
Number (%) of patients in each group with no requirement for additional airway interventions, *n* (%)	17 (43.6%)		26 (76.5%)		0.004
Number (%) of patients in each group with additional airway interventions applied during sedation					
(1) head lateral rotation	18 (46.2%)		1 (2.9%)		<0.001
(2) neck extension	8 (20.5%)		1 (2.9%)		0.032
(3) oral airway insertion	12 (30.8%)		5 (14.7%)		0.165
(4) nasal airway insertion	0 (0%)		1 (2.9%)		0.466
(5) jaw thrust maneuver	3 (7.7%)		5 (14.7%)		0.460

Numbers are median [IQR, interquartile range] or numbers of patients (percentage) in each group. 95% CI, 95% confidence interval. *p* value < 0.05 is considered significant.

**Table 4:** Polysomnography data.

	Conventional Group *n* = 36		JED Group *n* = 31		
	Median (IQR)	95% CI	Median (IQR)	95% CI	*p* value
Polysomnography analysis time (min)	77 (70.5–88.0)	72.0–81.7	82 (74.5–91.8)	77.6–90.4	0.280
AHI	9.3 (3.8–21.9)	6.1–17.9	4.5 (1.5–11.9)	1.7–7.6	0.015
Snoring time (min)	45.7 (29.7–56.0)	37.3–54.8	39.2 (23.2–57.3)	30.6–53.9	0.440
Relative snoring time per analysis (%)	63.8 (43.5–77.3)	53.5–69.8	46.3 (27.9–76.5)	39.8–70.9	0.228
Average SpO_2_	98.9 (98.3–99.4)	98.5–99.2	99.2 (98.4–99.9)	98.9–99.4	0.206

Numbers are median [IQR, interquartile range] or numbers of patients (percentage) in each group. 95% CI, 95% confidence interval. AHI, apnea-hypopnea index. *p* value < 0.05 is considered significant.

## Data Availability

The data presented in this study are available on request from the corresponding author.
